# Gene coexpression network analysis of fruit transcriptomes uncovers a possible mechanistically distinct class of sugar/acid ratio-associated genes in sweet orange

**DOI:** 10.1186/s12870-017-1138-8

**Published:** 2017-10-30

**Authors:** Liang Qiao, Minghao Cao, Jian Zheng, Yihong Zhao, Zhi-Liang Zheng

**Affiliations:** 1 Plant Nutrient Signaling and Fruit Quality Improvement Laboratory, National Citrus Engineering Research Center, Citrus Research Institute, Southwest University, Beibei, Chongqing, 400712 China; 2 Division of Biostatistics, Department of Child and Adolescent Psychiatry, New York University Langone Medical Center, New York, NY 10016 USA; 3 Department of Biological Sciences, Lehman College, City University of New York, Bronx, NY 10468 USA

**Keywords:** Citrus, Orange, Fruit, Acids, Sugars, Sugar/acid ratio, Transcriptome, Gene networks

## Abstract

**Background:**

The ratio of sugars to organic acids, two of the major metabolites in fleshy fruits, has been considered the most important contributor to fruit sweetness. Although accumulation of sugars and acids have been extensively studied, whether plants evolve a mechanism to maintain, sense or respond to the fruit sugar/acid ratio remains a mystery. In a prior study, we used an integrated systems biology tool to identify a group of 39 acid-associated genes from the fruit transcriptomes in four sweet orange varieties (*Citrus sinensis* L. Osbeck) with varying fruit acidity, Succari (acidless), Bingtang (low acid), and Newhall and Xinhui (normal acid).

**Results:**

We reanalyzed the prior sweet orange fruit transcriptome data, leading to the identification of 72 genes highly correlated with the fruit sugar/acid ratio. The majority of these sugar/acid ratio-related genes are predicted to be involved in regulatory functions such as transport, signaling and transcription or encode enzymes involved in metabolism. Surprisingly, only three of these sugar/acid ratio-correlated genes are weakly correlated with sugar level and none of them overlaps with the acid-associated genes. Weighted Gene Coexpression Network Analysis (WGCNA) has revealed that these genes belong to four modules, Blue, Grey, Brown and Turquoise, with the former two modules being unique to the sugar/acid ratio control.

**Conclusion:**

Our results indicate that orange fruits contain a possible mechanistically distinct class of genes that may potentially be involved in maintaining fruit sugar/acid ratios and/or responding to the cellular sugar/acid ratio status. Therefore, our analysis of orange transcriptomes provides an intriguing insight into the potentially novel genetic or molecular mechanisms controlling the sugar/acid ratio in fruits.

**Electronic supplementary material:**

The online version of this article (10.1186/s12870-017-1138-8) contains supplementary material, which is available to authorized users.

## Background

Fleshy fruits contain various sugars and organic acids that are among the most important considerations for improving sensory traits such as taste and flavor. Fruit sweetness is determined by the levels of sugars and acids and their ratio [1–3]. The major sugars in fruits include sucrose, glucose and fructose, and malate and citrate are two major types of organic acids in fruits. Although the genes encoding enzymes involved in sugar or acid metabolism have long been proposed to control fruit sugar or acid levels, the intensive molecular biology studies so far have only led to the demonstration of two acid metabolism-related genes (a specific isoform of aconitase and a malate dehydrogenase) in the control of fruit acidity [4, 5].

To identify additional genes responsible for controlling fruit sweetness, many genetic, molecular and transcriptomic studies have been focused on the transport, storage and degradation of sugars and acids in both climacteric fruits such as tomato [4–9], and non-climacteric fruits such as citrus [10–15]. Recent genetic evidence has revealed important roles of the malate transporter MdMa1 [16], biochemically unknown CmPH gene [17] and the MdMYB transcription factor [18] in controlling apple or melon fruit acidity. Other genetic loci are yet to be cloned, and for many other genes correlated with sugar and/or acid levels, their functions in the control of fruit sweetness remain to be demonstrated by convincing genetic evidence.

Surprisingly, very few studies have examined the sugar/acid ratios. Mathematically, the sugar/acid ratio can be determined by changing the sugar or acid level alone or altering both sugar and acid levels in opposite directions. A recent genetic study showed that antisense suppression of a malate dehydrogenase gene *MDH2* in tomato fruits increased the malate level but also led to a reduction in soluble sugar content [4], thus greatly increasing the sugar/acid ratio. Therefore, even a mild change in sugar and acid levels in opposite directions may lead to a significant alteration of the sugar/acid ratio. Such a tight link between sugar and acid metabolism has led to the argument that the sugar/acid ratio can only be regarded as a subjective parameter to evaluate the consumer’s perception of fruit sweetness rather than a horticultural trait. However, results from a genetic analysis involving a regular melon cultivar and its acidic melon bred line suggest distinct genetic variants controlling sugar content, acid content and the sugar/acid ratios [19]. Therefore, examining the genes or genetic variants correlated with the sugar/acid ratios has a potential to facilitate the identification of additional regulatory genes involved in the control of fruit sweetness.

In this study, we use citrus as a model to reanalyze the fruit transcriptome data recently published from our group [14]. Citrus, representing one of the largest fresh fruit and juice industry in the world, has the potential for use as an economically important tree fruit model organism to study non-climacteric fruit development. Citrus has a relatively small genome size (approximately 367 Mb), and efficient genetic transformation techniques have been developed for various citrus cultivars or varieties [20, 21]. In our prior study, we have identified a total of 39 genes strongly correlated with fruit acidity using the four varieties of sweet orange, ranging from acidless (Succari) to low acid (Bingtang) and normal acid (Newhall and Xinhui), which contain similar acid levels at Stage I (cell division; 45 days post anthesis/DPA) but exhibit differing acidity at Stage II (cell expansion; 142 DPA). Although these varieties were originally designed for the acid-related transcriptomic studies, we have found that they exhibit different sugar/acid ratios. Because there has been no transcriptomic study aiming at the genes specifically involved in the control of fruit sugar/acid ratio, we believe that reanalysis of this data will provide important insights. Towards this, we have identified a total of 72 genes strongly correlated with the sugar/acid ratios, and yet most of them do not correlate with either sugar or acid contents alone. Furthermore, since our first report of using systems biology approach in unraveling the citrus host response to the attack by one of the most constructive disease Huanglongbing [22], the gene coexpression-based approach has been increasingly used by us and other groups to further understand the gene networks involved in Huanglongbing pathogenesis and fruit development or predict gene annotations [14, 23–25]. Therefore, in this study we also use the network approach to analyze the fruit transcriptome data, leading us to conclude that these 72 sugar/acid ratio-related genes belong to four modules of the fruit development gene coexpression network, two of which have no overlap with the sugar- and acid-related network modules. Taken together, our results have uncovered the genes and coexpression modules uniquely associated with the control of sugar/acid ratio in expanding fruits.

## Results

### Analysis of the fruit sugar/acid ratios in four sweet orange varieties with differing fruit acidity

**Fig. 1:**
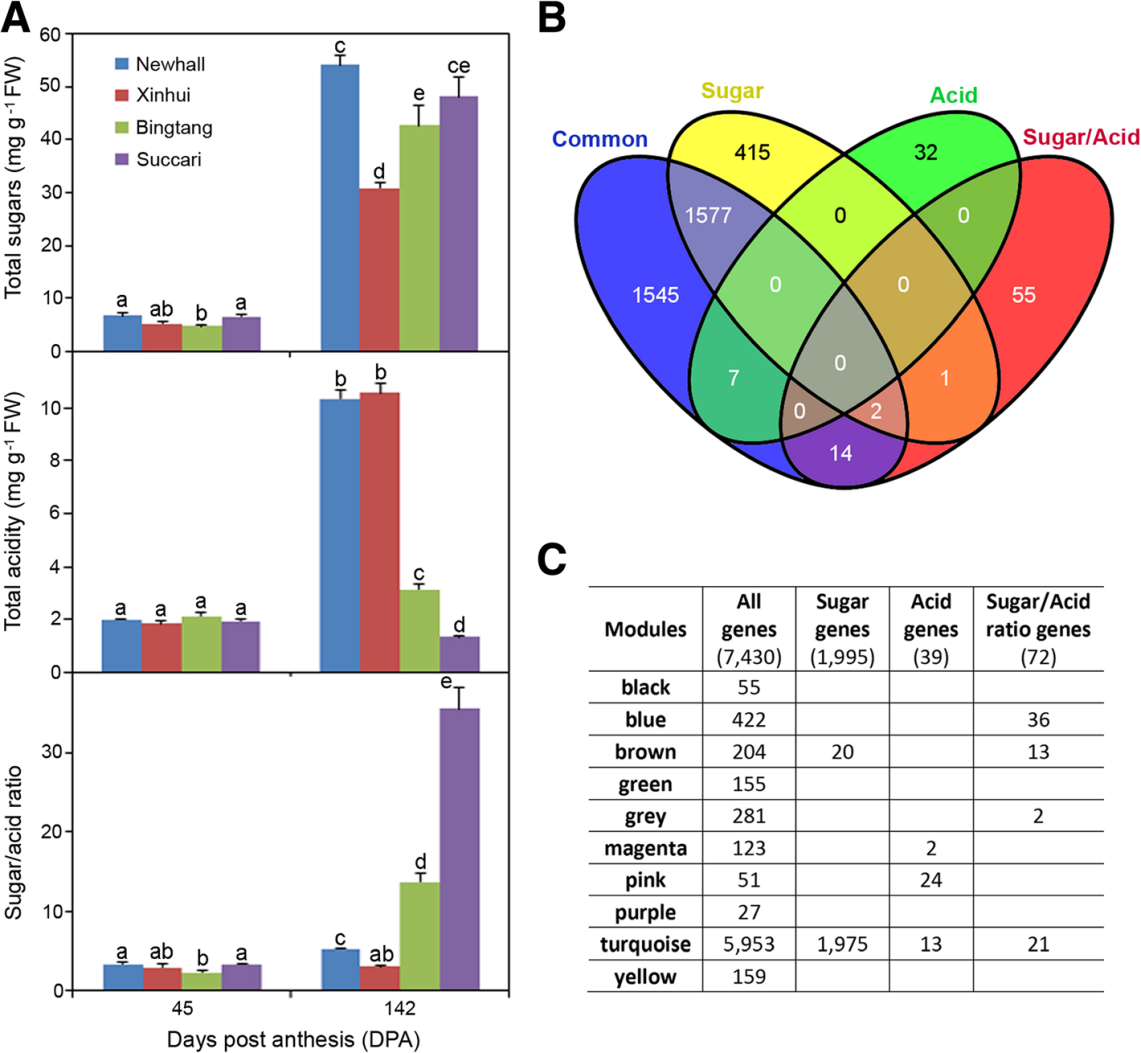
Comparison of sugar/acid ratios in four different sweet orange varieties and of genes and modules associated with sugar, acid and sugar/acid ratios. (**a**) Comparison of sugar and acid levels and sugar/acid ratios in different sweet orange varieties. Sugar and acid levels were adapted from a prior manuscript [14], and the sugar/acid ratios were calculated using the sugar and acid levels in each fruit for each variety. Values are means and SE of three fruits. Different letters above the column indicates a significant difference (*p* < 0.05). (**b**) Venn diagram of overlapping genes correlated with sugar and acid levels and sugar/acid ratios. “Common” refers to the genes commonly regulated from 45 to 142 DPA in all varieties. (**c**) Comparison of the number of genes associated with sugar, acid and sugar/acid ratio in each of the gene co-expression modules derived from WGCNA

To compare the fruit sugar/acid ratios in the four varieties of sweet orange, Newhall, Xinhui, Bingtang and Succari, we re-analyzed the fruit sugar and acid accumulation data reported previously [14]. At 45 DPA, the four varieties had sugar/acid ratios of 2.3–3.3, but because of similar increase in sugar levels and different acid levels at 142 DPA, they exhibited different sugar/acid ratios at 142 DPA (Fig. 1a). While Xinhui fruits had the same sugar/acid ratio, Newhall showed a slight increase of the ratio from 3.3 to 5.2. However, the low acid variety Bingtang and the acidless variety Succari had much higher sugar/acid ratios at 142 DPA (13.7 and 35.6, respectively). Therefore, it appears that the four varieties exhibit opposite trends for acidity and sugar/acid ratio.

### Identification and analysis of genes correlated with the sugar/acid ratio

To identify the candidate genes that are strongly correlated with the fruit sugar/acid ratio in sweet orange fruits, we performed a correlation study between sugar/acid ratio and expression level of 7430 genes which are differentially expressed between 45 and 142 DPA in any of the four varieties reported previously [14]. As a result, we identified a total of 72 genes which showed strong correlations with sugar/acid ratio, with Pcc > = 0.80 or <= −0.80 and a minimal false discovery rate (FDR) of 3.5E-04 (Table 1). Among those 72 genes, only 10 (14%) are negatively correlated with sugar/acid ratio, while all others have positive correlations. The three genes with the strongest correlation (Pcc = 0.90 or 0.91) are Cs1g14800 encoding a Leucine-rich repeat (LRR) receptor-like serine/threonine protein kinase, Cs1g03610 predicted to encode a malate dehydrogenase and Cs7g06285 with no Arabidopsis homolog revealed. Results of qPCR-based expression for six genes showed that gene expression levels measured by qPCR and RNA seq are highly correlated, with Pcc ranging from 0.93 to 0.99 and *p*-values of <0.001 (Table 2). This indicates that our RNA sequencing data is reliable for use in analysis of genes involved in the control of sugar/acid ratio.

**Table 1:** A list of genes strongly correlated with the sugar/acid ratio in orange fruits

CsGID	Pcc	FDR	Module	AtGID	Arabidopsis gene description
Transport (9)					
Cs1g13320	0.87	1.3E-05	turquoise	At1g15460	BOR4, Requires High Boron 4
Cs1g25820	0.87	1.4E-05	blue	At5g66110	HIPP27, heavy metal associated isoprenylated plant protein 27
Cs2g06990	0.89	8.7E-06	blue	At4g05120	FUR1, FUDR Resistant 1
Cs4g20090	0.89	6.7E-06	brown	At4g32650	KAT3, potassium channel in *Arabidopsis thaliana* 3
Cs5g17210	0.81	2.1E-04	blue	At1g17810	Beta-TIP, beta-tonoplast intrinsic protein
Cs5g24670	−0.87	1.4E-05	blue	At5g48490	Putative lipid transfer/seed storage protein
Cs5g31010	0.83	1.5E-04	blue	At3g22600	Non-specific lipid-transfer/seed storage protein
Cs9g18100	0.80	3.2E-04	turquoise	At5g53130	CNGC1, cyclic nucleotide gated channel 1
orange1.1 t01769	0.82	1.7E-04	blue	At3g62150	ABCB21, ATP-binding cassette B21
Signaling (6)					
Cs1g01170	0.86	3.0E-05	blue	At4g23160	CRK8, cysteine-rich RLK (RECEPTOR-like protein kinase) 8
Cs1g14800	0.90	5.0E-06	blue	At3g47570	probable LRR receptor-like serine/threonine protein kinase
Cs2g02680	0.88	8.7E-06	brown	At3g21630	CERK1, chitin elicitor receptor kinase 1
Cs3g07510	0.80	3.2E-04	blue	At1g03430	AHP5, histidine-containing phosphotransfer factor 5
Cs6g02710	0.81	2.3E-04	turquoise	At1g16120	WAKL1, wall associated kinase-like 1
Cs6g05010	0.80	3.5E-04	blue	At3g47570	probable LRR receptorlike serine/threonineprotein kinase
Transcription (7)					
Cs3g01830	0.86	3.0E-05	brown	At3g54460	F-box family protein
Cs5g03630	0.83	1.4E-04	brown	At5g23750	remorin like
Cs6g03950	−0.80	3.2E-04	turquoise	At2g37260	TTG2, Transparent testa Glabra 2, WRKY
Cs7g27620	0.85	4.1E-05	brown	At1g07900	LBD1, LOB domain-containing protein 1
Cs9g08500	0.80	3.2E-04	turquoise	At5g43630	TZP, tandem zinc knuckle protein
orange1.1 t00185	0.83	1.2E-04	blue	At4g00050	UNE10, unfertilized embryo sac 10, bHLH
orange1.1 t00294	0.80	3.2E-04	brown	At4g16110	RR2/ARR2, response regulator 2
Protein degradation (3)					
Cs8g05200	0.80	3.2E-04	blue	At1g47128	RD21A, responsive to dehydration 21A
orange1.1 t00281	0.88	8.7E-06	brown	At4g00230	XSP1, xylem serine peptidase 1
orange1.1 t02370	0.83	1.2E-04	blue	At2g31980	CYS2, Phytocystatin 2
Development (2)					
Cs4g15700	0.80	3.0E-04	turquoise	At4g29860	EMB2757, Embryo Deffective 2757
orange1.1 t02243	0.83	1.4E-04	blue	At5g06760	LEA4–5, Late Embryogenesis Abundant 4–5
Stress response (4)					
Cs5g18450	0.82	1.7E-04	turquoise	At5g17680	Leucine rich repeat-containing protein
Cs5g19950	0.85	4.3E-05	turquoise	At5g17680	TMV resistance protein N-like
Cs7g32260	0.80	3.5E-04	turquoise	At5g59720	HSP18.2, heat shock protein 18.2
orange1.1 t01829	0.84	7.8E-05	turquoise	At5g17680	TMV resistance protein N-like
Metabolism (20)					
Cs1g03610	0.91	4.9E-06	blue	At5g43330	c-NAD-MDH2, cytosolic-NAD-dependent malate dehydrogenase 2
Cs1g10530	0.81	2.3E-04	turquoise	At2g18950	HPT1, homogentisate phytyltransferase 1
Cs1g25210	−0.84	9.1E-05	brown	At2g18020	EMB2296, embryo defective 2296, ribosomal protein
Cs2g27550	0.80	3.2E-04	blue	At3g01570	Oleosin family protein
Cs3g12560	0.81	2.7E-04	blue	At1g54740	ribosomal protein
Cs3g24700	0.81	2.3E-04	brown	At5g42740	PGI, glucose-6-phosphate isomerase, cytosolic 1
Cs4g14130	0.81	2.3E-04	blue	At2g45550	CYP76C4, cytochrome P450, family 76, subfamily C, polypeptide 4
Cs5g18850	0.82	1.5E-04	blue	At1g71250	GDSL-motif esterase/acyltransferase/lipase
Cs5g20010	0.83	1.2E-04	turquoise	At1g35190	Hyoscyamine 6-dioxygenase
Cs7g13310	−0.83	1.2E-04	turquoise	At1g68530	KCS6, 3-ketoacyl-CoA synthase 6
Cs7g30920	0.81	2.2E-04	blue	At2g40170	GEA6/Em6, Late embryogenesis abundant 6
Cs7g31620	0.80	3.0E-04	blue	At2g38080	IRX12, Irregular Xylem 12
Cs8g06880	−0.84	1.1E-04	grey	At4g36750	Minor allergen Alt a, lipid metabolism
Cs9g06700	0.86	3.0E-05	brown	At1g31690	Copper amine oxidase family protein
Cs9g07750	−0.89	7.0E-06	brown	At3g22890	APS1, ATP sulfurylase 1
Cs9g13750	0.81	2.3E-04	blue	At5g07475	Cupredoxin superfamily protein; copper ion binding
Cs9g17670	0.81	2.3E-04	grey	At4g23420	short chain dehydrogenase/reductase (SDR)
orange1.1 t00612	0.82	2.0E-04	turquoise	At5g66460	MAN7, endo-beta-mannase 7
orange1.1 t02858	0.81	2.7E-04	blue	At1g79640	serine/threonine protein kinase, putative
orange1.1 t03587	0.83	1.2E-04	turquoise	At1g17020	SRG1, senescence-related gene 1
Unknown function (11)					
Cs1g20290	0.80	3.0E-04	blue		
Cs1g20300	0.80	3.2E-04	blue		
Cs1g23800	0.80	3.2E-04	blue	At2g18540	vicilin GC72A like, cupin
Cs1g24590	0.81	2.3E-04	blue		
Cs2g06500	−0.82	2.0E-04	turquoise	At1g11090	MGL, Monoglyceride lipase
Cs2g07220	−0.88	1.2E-05	blue	At3g13160	pentatricopeptide repeat-containing protein
Cs2g20110	0.88	8.7E-06	blue		
Cs3g17860	0.81	2.1E-04	brown		queuine tRNA ribosyltransferase like
Cs5g05940	0.80	3.2E-04	blue		
Cs5g06080	0.88	9.3E-06	turquoise	At5g50170	C2 and GRAM domain-containing protein
Cs5g20400	0.81	2.2E-04	turquoise		
Cs5g23250	0.81	2.1E-04	turquoise	At5g67550	
Cs6g16160	−0.89	7.0E-06	blue	At4g24380	hypothetical protein SORBIDRAFT_02g043510
Cs7g06285	0.90	4.9E-06	brown		
Cs7g23240	0.86	2.9E-05	blue	At5g04830	hypothetical protein ARALYDRAFT_487267
Cs7g25170	−0.82	1.5E-04	turquoise		
Cs7g30380	0.83	1.5E-04	blue	At2g40390	
Cs8g10300	0.84	1.1E-04	blue		
Cs8g18390	−0.80	3.0E-04	turquoise		
Cs8g19210	0.82	2.0E-04	blue		
orange1.1 t01135	0.84	7.8E-05	blue		hypothetical protein ARALYDRAFT_487267

A total of 72 citrus genes are highly correlated with the sugar/acid ratios in orange fruits of four varieties, with a Pearson correlation coefficient (Pcc) of above 0.80 or below −0.80 and an adjusted *p*-value (FDR, false discovery rate) of larger than 1.0E-04. CsGID, Cs gene ID. The number of genes for individual biological process is indicated in parenthesis and the module to which the genes belong is inidcated. The most closely related homologs of Arabidopsis gene for each citrus gene is presented as AtGID (At gene ID), with Arabidopsis gene description shown. Absence of AtGID indicates no Arabidopsis homolog for CsGID identified

**Table 2:** Pearson correlation analysis of sugar/acid ratio-related gene expression levels detected by RNA sequencing and qPCR

Genes	Methods	45 DPA				142 DPA				Pcc	*p*-value
		Newhall	Xinhui	Bingtang	Succari	Newhall	Xinhui	Bingtang	Succari		
Cs1g03610 (malate dehydrogenase)	RNAseq	0.4	0.4	0.3	0.6	0.6	0.4	0.6	3.8	0.99	1.3E-08
	qPCR	1.0	1.4	0.8	1.6	1.3	1.4	1.4	10.3		
Cs5g03630 (remorin like)	RNAseq	0.8	1.3	0.5	1.3	1.8	1.8	1.3	7.1	0.99	1.6E-07
	qPCR	1.0	1.3	0.8	1.4	2.3	2.6	2.2	11.2		
Cs5g20010 (Hyoscyamine 6dioxygenase)	RNAseq	0.3	0.2	0.1	0.1	0.8	0.3	1.5	2.0	0.99	3.1E-07
	qPCR	1.0	0.8	0.6	0.4	4.2	1.0	7.8	11.8		
Cs5g24670 (lipid transfer)	RNAseq	47.2	45.9	58.2	93.3	39.4	42.8	25.5	4.6	0.93	7.1E-04
	qPCR	1.0	1.0	1.0	2.1	1.2	1.2	0.6	0.2		
Cs6g16160 (unknown)	RNAseq	78.3	76.9	73.4	29.8	148.0	63.9	17.3	2.3	0.97	4.6E-05
	qPCR	0.8	1.3	0.5	1.3	1.8	1.8	1.3	7.1		
Cs7g32260 (HSP18.2)	RNAseq	0.4	0.4	0.4	0.1	5.3	5.7	4.5	109.3	0.99	6.5E-11
	qPCR	1.0	0.8	0.6	0.6	32.9	24.0	16.9	776.8		

Expression levels for six genes which were determined to be differentially regulated between different varieties by RNA sequencing (RNA seq) were validated by using quantitative PCR (qPCR) analysis. Values are the means of RNA seq data (RPKM) or qPCR data (with the value for Newhall at 45 DPA set as 1 after normalization to the *Actin* control) from three biological replicates. DPA, days post anthesis; Pcc, Pearson correlation coefficient

Gene ontology (GO) analysis and Arabidopsis gene homolog prediction result showed that 11 of 72 sugar/acid ratio-correlated genes have unknown functions, 20 likely function in metabolism, and the remaining 41 genes are involved in regulatory functions including transport, signaling, transcription, degradation, stress response and development (Table 1).

The 20 genes in the metabolism GO category encode a wide array of enzymes involved in various aspects of metabolism, including secondary metabolism (Cs5g20010/ hyoscyamine 6-dioxygenase, Cs7g13310/KCS6, Cs7g31620/IRX12 and orange1.1 t03587/SRG1), lipid metabolism (Cs2g27550 and Cs8g06880), protein synthesis (Cs1g25210/EMB2296, and Cs3g12560, both being ribosomal proteins), primary carbon metabolism (Cs3g24700/PGI, and Cs1g03610/MDH2), and sulfate metabolism (Cs9g07750/APS1). Of particular note, Cs3g24700 is orthologous to At5g42740-encoded cytosolic glucose-6-phosphate isomerase (PGI) involved in glycolysis [26], and Cs1g03610, the most strongly sugar/acid ratio-correlated gene, is most closely related to the Arabidopsis cytosolic-NAD-dependent malate dehydrogenase (c-NAD-MDH2), raising the possible involvement of malate metabolism and glycolysis in the fruit sugar/acid ratio control. The role of Cs9g07750, which is predicted to encode APS1, an enzyme involved in sulfate assimilation to Cys [27], in fruit sugar and acid metabolism remains unclear. The observation that sulfur metabolism cross-talks with the carbohydrate and nitrogen status [28] might indicate a possible role for modulation of Cs9g07750/APS1 expression in sensing of or response to sugar level.

As in the case of acid-related genes [14], a considerable proportion (19) of the sugar/acid ratio-correlated genes are involved in transport, transcription or protein degradation. Among nine genes in the transport category, two (Cs5g24670 and Cs5g31010) are predicted to act in lipid transfer in the seed storage process. Cs2g06990 is closely related to FUR1, a nucleoside transporter [29], while orange1.1 t01769 is similar to ATP-binding cassette B21, which is involved in auxin efflux and influx [30]. Cs1g13320 is similar to an efflux-type borate transmembrane transporter [31], Cs4g20090 is closely related to potassium channel protein KAT3, Cs9g18100 is predicted to encode a cyclic nucleotide gated channel (CNGC1), and both Cs1g25820 and Cs5g17210 show high similarity to Arabidopsis metal ion transporter. Regarding the three genes involved in protein degradation, two of them (Cs8g05200 and orange1.1 t02370) are putative cysteine proteases and the other one (orange1.1 t00281) may act as a serine peptidase. With regard to the seven genes in the category of transcription, they belong to distinct family of transcriptional regulators, such as F-box family (Cs3g01830), WRKY family (Cs6g03950), bHLH family (orange1.1 t00185), tandem zinc family (Cs9g08500), remornin-like (Cs5g03630), LBD family (Cs7g27620), and ARR family (orange1.1 t00294). In Arabidopsis, TTG2 is involved in proanthocyanidin synthesis and cell size control [32]. ARR2 has been shown to act in response to cytokinin and ethylene and in the expression of nuclear genes encoding components of mitochondrial complex I [33, 34]. Overall, none of these three categories of proteins has been shown to be involved in regulation of sugar or acid synthesis, transport or response.

The sugar/acid ratio-correlated genes also have two GO categories that were not enriched for the acid-correlated genes: signaling and stress response. Interestingly, five of six signaling-related genes encode receptor-like kinases (RLK), such as Cs1g01170/CRK8 and Cs6g02710/WAKL1, and the remaining one is Cs3g07510/AHP5. In Arabidopsis, CRK8 and CERK1 have been shown to act in defense response [35, 36] and expression of *CRK8* could be modulated by hormones [35]. AHP5, one of the six Arabidopsis histidine phosphotransfer proteins (AHPs), has a redundant function as positive regulators of cytokinin signaling [37]. Concerning the category of stress response, three of four genes (Cs5g18450, Cs5g19950, orange1.1 t01829) are involved in biotic stress response, while the fourth one, Cs7g32260/HSP18.2, is predicted to act in abiotic stress response.

### Comparison of sugar/acid ratio-correlated genes with sugar- or acid-associated genes

As mathematically the sugar/acid ratio could be affected by changes in levels of sugar alone, acid alone or both, we decided to assess whether any of the 72 sugar/acid ratio-correlated genes has overlap with the set of 39 acid-correlated genes. Surprisingly, analysis using Venn diagram showed that none of the acid- and sugar/acid ratio-correlated genes overlaps (Fig. 1b).

We then analyzed the degree of overlap between the sugar/acid ratio-correlated genes and the sugar-correlated genes. We were surprised that 4166 out of 7430 differentially expressed genes exhibited strong correlations with sugar level, with Pcc above 0.8 or below −0.8 (Additional file 1). Thus, we used a higher Pcc cutoff (+/−0.9) for the prediction of sugar-correlated genes, with a total of 1995 genes (Additional file 1). Comparison of these 1995 genes and 72 sugar/acid ratio-related genes revealed that only three genes (Cs1g10530, Cs8g18390 and Cs9g08500) are strongly correlated with both sugar level and sugar/acid ratio (Fig. 1b). Nine additional sugar/acid ratio-related genes also had Pcc with sugar between 0.8 and 0.9 (Additional file 2). This indicates that the majority of the sugar/acid ratio-correlated genes do not strongly correlate with sugar level. Taken together, our comparative analysis of sugar-, acid- and sugar/acid ratio-correlated genes strongly indicates that at least in these four sweet orange varieties a unique subset of genes might be involved in the control of fruit sugar/acid ratio independent of sugar and acid accumulation alone.

To reveal whether the control of sugar/acid ratio involves distinct gene coexpression modules, we respectively mapped the subsets of acid-, sugar- and sugar/acid-correlated genes into the 10 modules present in the gene coexpression network reported in our prior work [14]. Results showed that among the 1995 sugar-correlated genes, 1975 belong to the Turquoise module and 20 are classified to the Brown module; however, the sugar/acid ratio-correlated genes can be grouped into four modules, Blue, Grey, Brown and Turquoise (Fig. 1c). Compared to the acid-related gene modules, the sugar/acid ratio-correlated genes contain three distinct modules, Blue, Brown and Grey. Taken together, the sugar/acid ratio-related genes possess two unique modules, Blue and Grey. The Blue module is the largest one, containing 36 out of 72 sugar/acid ratio genes, while the Grey module has only two genes (Fig. 1c; Table 1). Thus, the Blue module might represent a distinct subnetwork for the control of fruit sugar/ acid ratio.

### Construction and analysis of distinct module-based sugar/acid ratio gene subnetworks

To provide a systems view of gene networks for the control of sugar/acid ratio in orange fruits, we constructed the subnetworks based on the four distinct gene coexpression modules, Blue, Brown, Grey and Turquoise (Fig. 1c). The subnetworks were built using the module-specific subset of sugar/acid ratio-related genes as seed nodes to extract the gene coexpression network previously constructed by WGCNA analysis of 7430 genes [14]. Except for the Grey module with two genes, all other modules have genes present in the network and are analyzed below.

**Fig. 2:**
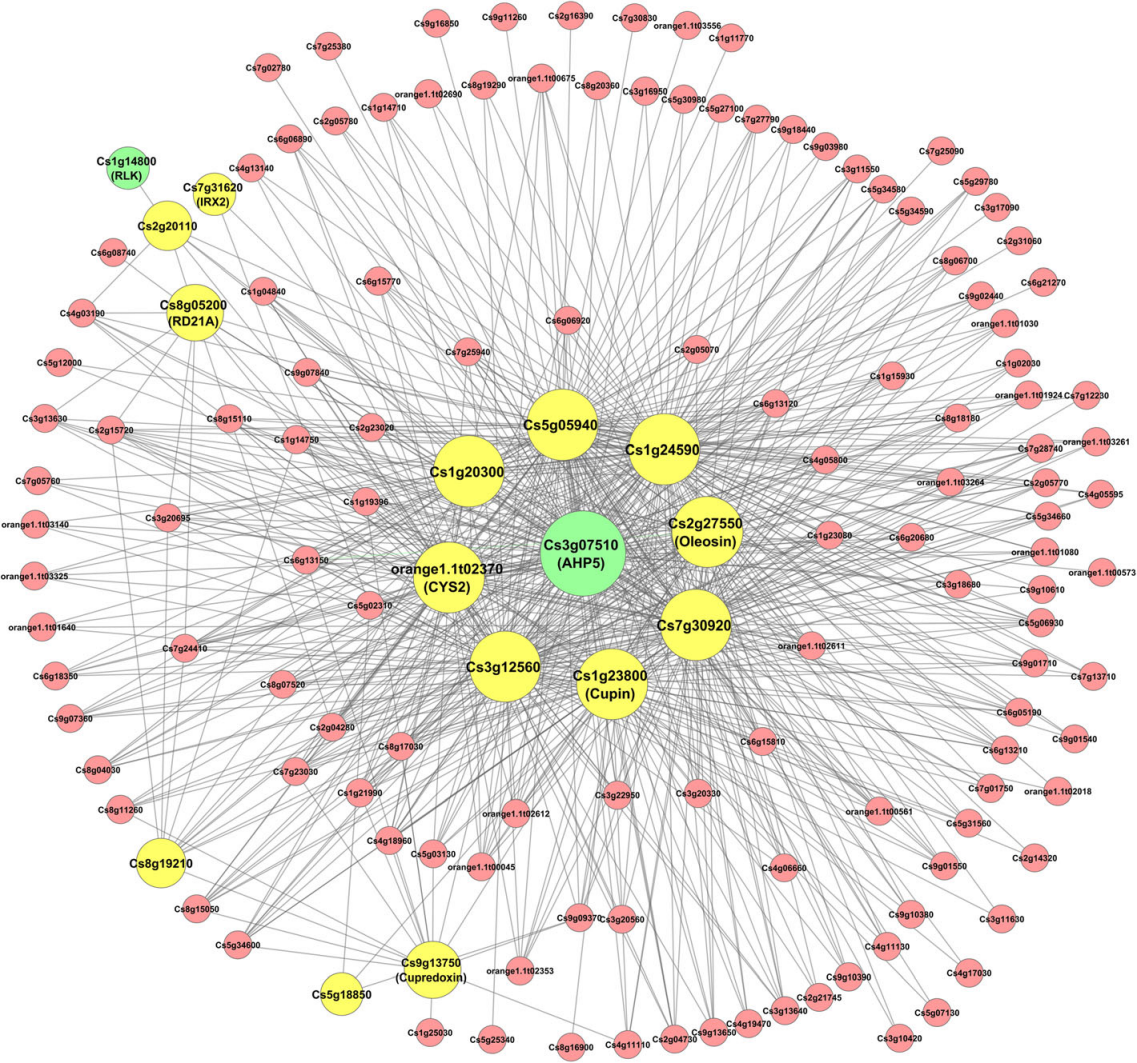
The Blue module-based sugar/acid ratio-associated gene subnetwork. The subnetwork constructed by using the sugar/acid ratio-correlated genes from the blue module as seed nodes to extract the weighted gene coexpression network of all 7430 differentially expressed genes with an edge weight cutoff of 0.6 is visualized by Cytoscape. Two seed node genes involved in signal transduction are coded in green, and all other seed node genes coded in yellow

The Blue module-based subnetwork contains 16 of 36 sugar/acid ratio-correlated genes (coded in yellow and green), with a total of 142 nodes and 689 edges (Fig. 2). In this module, nine of sugar/acid ratio genes represent large hubs, with five of them having more than 90 edges or interactions, including Cs3g07510/AHP5, Cs1g24590 (unknown function), Cs7g30920/GEA6, Cs3g12560 (a ribosomal protein involved in protein synthesis) and Cs5g05940 (unknown function). Four other mid-size hubs are Cs1g23800 (a functionally unknown Cupin-like protein), orange 1.1 t02370/CYS2 (involved in protein degradation), Cs1g20300 (unknown function), and Cs2g27550/Oleosin (involved in lipid metabolism). The Arabidopsis homologs for most of these hub genes have not been tested regarding their physiological functions. Cs7g30920 is most similar to Arabidopsis GEA6 or Em6, a group 1 LEA gene that is involved in response to abscisic acid and is predicted to act in metabolism during seed development [38]. Cs3g07510/AHP5 and a RLK gene encoded by Cs1g14800 are involved in signal transduction. The finding that Cs3g07510/AHP5 acts as a large hub indicates a potentially crucial role of cytokinin signaling in the sugar/acid ratio subnetwork.

**Fig. 3:**
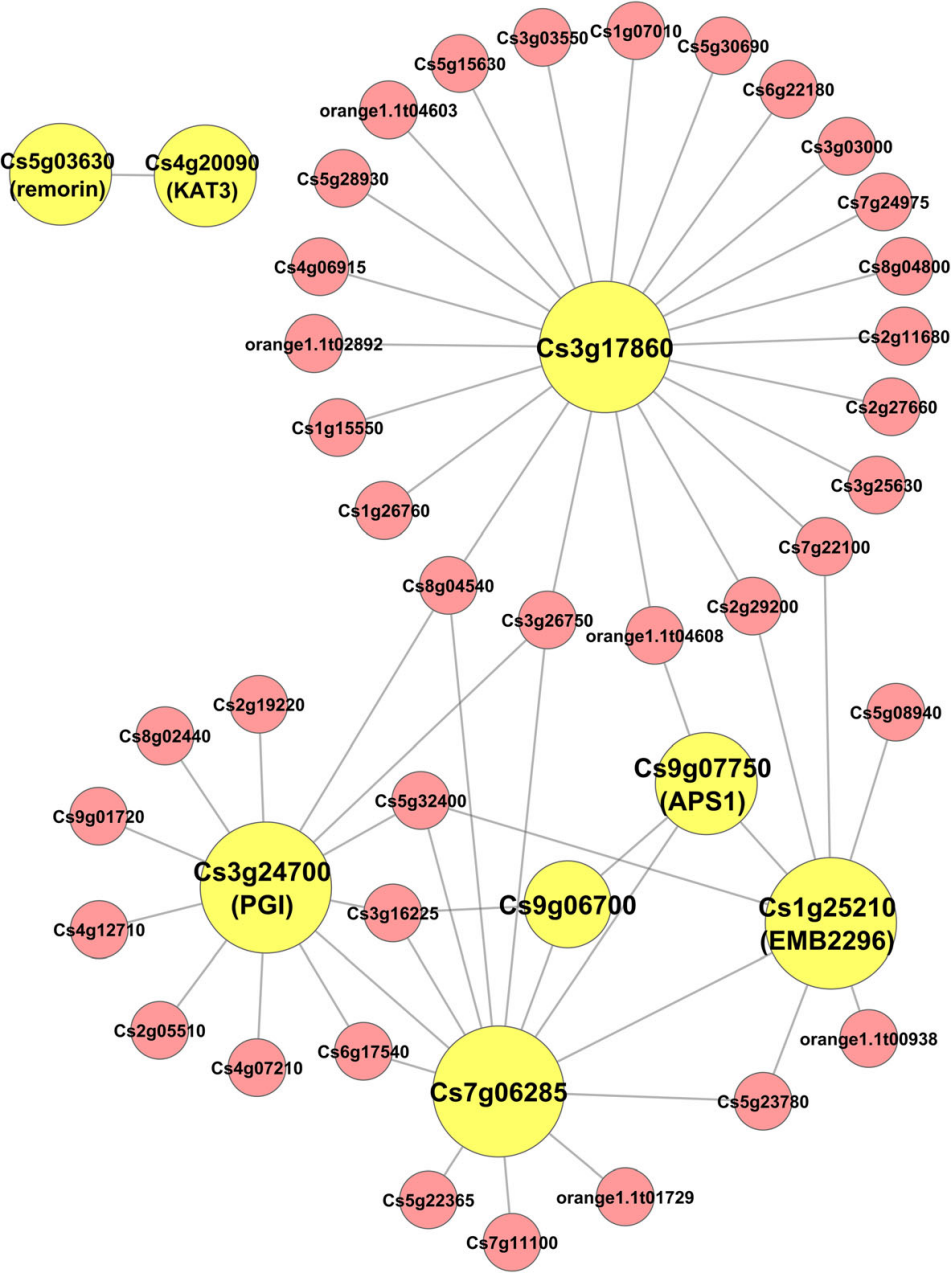
The Brown module-based sugar/acid ratio gene subnetwork. The sugar/acid ratio-correlated genes from the brown module are used as seed nodes to extract the weighted gene coexpression network as described in Fig. 2, resulting in the subnetwork with an edge weight cutoff of 0.2. The seed node genes present in the subnetwork are coded in yellow

The Brown module-based subnetwork has 45 nodes, including eight of the 13 sugar/acid ratio-correlated genes, and 57 edges (Fig. 3). In this module, Cs5g03630/remorin and Cs4g20090/KAT3 only interact with each other. It remains to be determined whether the interaction between remornin-like transcriptional regulator and the potassium channel KAT3 has any physiological or metabolic relevance in the sugar/acid ratio control. In the mid-sized subnetwork which contains all other nodes and edges, two hubs, Cs3g17860 and Cs7g06285, do not have their orthologs identified in Arabidopsis. The other hubs, Cs3g24700/PGI and Cs1g25210/EMB2296, together with Cs9g07750/APS1, are involved in metabolism, indicating a potential role of metabolic regulation in the Brown module-based sugar/acid ratio control subnetwork.

**Fig. 4:**
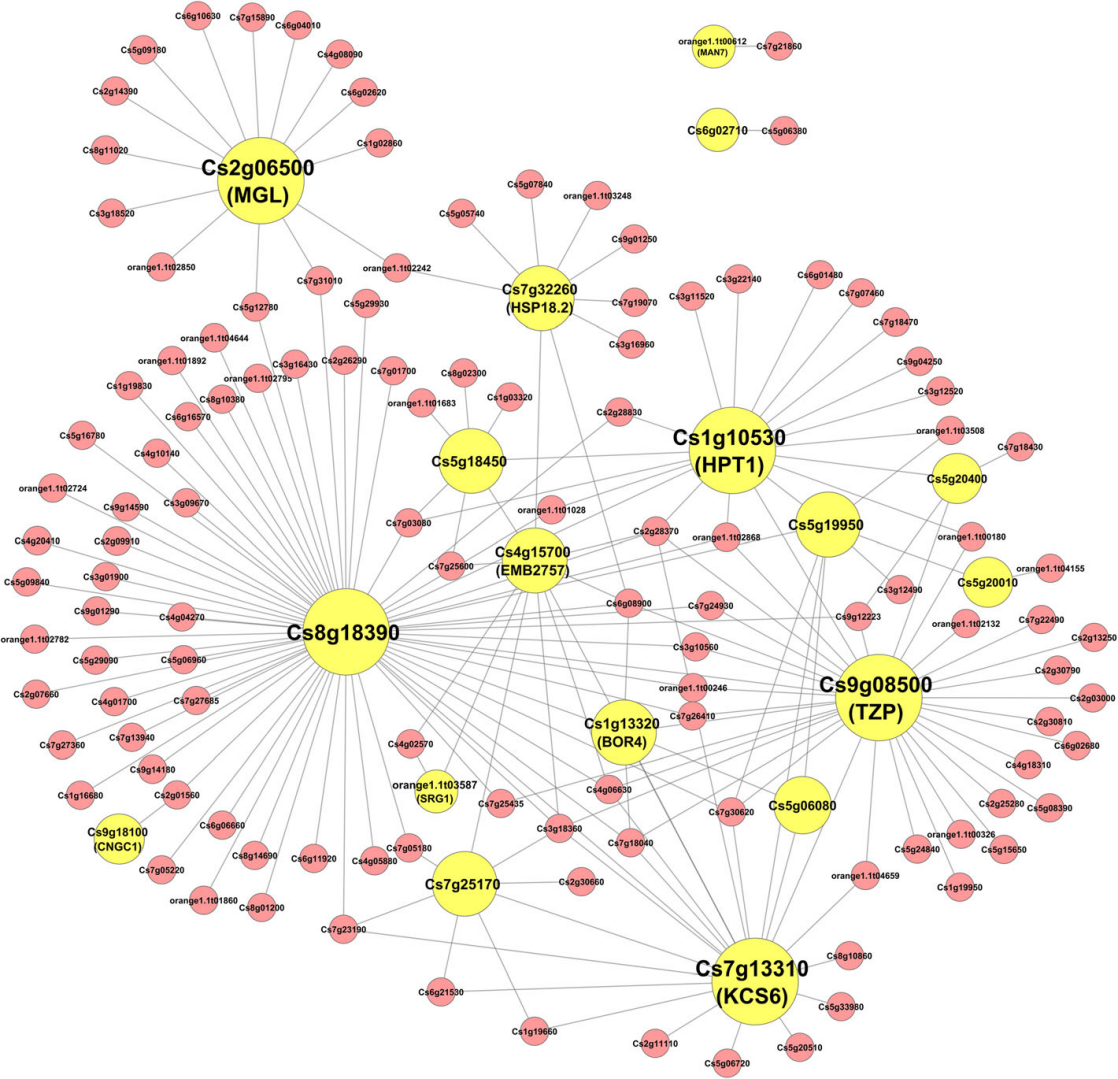
The Turquoise module-based sugar/acid ratio gene subnetwork. The subnetwork is constructed by extracting the weighted gene coexpression network using the sugar/acid ratio-correlated genes belonging to the Turquoise module as seed nodes, with an edge weight cutoff of 0.3. The seed node genes present in the subnetwork are coded in yellow

For the Turquoise module which contains 21 sugar/acid ratio-correlated genes, 18 of them are present in the subnetwork. This subnetwork has 137 nodes and 472 edges (Fig. 4). The largest hub Cs8g18390 does not have any Arabidopsis homolog, while all other hubs have their orthologs present in Arabidopsis, including a transcription factor gene Cs9g08500/TZP, two genes involved in metabolism (Cs1g10530/HPT1 and Cs7g13310/KCS6), a heat shock protein HSP18.2/Cs7g32260, an embryo development-related gene Cs4g15700/EMB2757, and the Cs2g06500-encoded putative monoglyceride lipase gene MGL. Besides HPT1 and KCS6, the two hub genes involved in metabolism, three other genes (Cs5g20010, orange1.1 t00612 and orange1.1 t03587) in the subnetwork are also related to metabolism, indicating that metabolism might have a critical role in in this Turquoise module-based sugar/acid ratio subnetwork. In addition, two genes related to transport (Cs1g13320/BOR4 and Cs9g18100/CNGC1) are also present in the subnetwork.

## Discussion

Despite the well-recognized contribution of the sugar/acid ratio in determination of the fruit sweetness trait, except for one genetic study in melon [19], none of the prior large-scale studies in both gene-trait association mapping or gene expression-trait correlation in tomato and citrus fruits [7–9, 11, 13–15] has attempted to address the possible relationship between genes and the sugar/acid ratio. Using the four sweet orange varieties with dramatic variations in fruit acidity and very small difference in sugar levels at 142 DPA, together with gene coexpression analysis, we have identified a subset of genes belonging to distinct modules that are strongly associated with the sugar/acid ratio.

Among 72 genes correlated with the sugar/acid ratio, two groups of genes are worth of further discussion here. The first group includes malate metabolism- and glycolysis-related genes, Cs1g03610/MDH2 and Cs3g24700/PGI. Given the predicted role of the cytosolic NAD-MDH2/Cs1g03610 in converting malate to pyruvate in the cytosol followed by glycolysis, perhaps this pathway is involved in slightly reducing citrate accumulation, contributing to the alteration of sugar/acid ratio. Alternatively, malate metabolism has been shown to be involved in the control of sugar accumulation [4]. Therefore, it remains a possibility that increasing orange *MDH2* gene expression would lead to enhanced malate metabolism, and subsequently sugar level may be slightly increased and acid level slightly reduced, leading to the increase of sugar/acid ratio. Future research directly testing this MDH gene, which exhibits the strongest correlation with the sugar/acid ratio in expanding orange fruits, will provide genetic evidence regarding the determination of sugar/acid ratio.

The second group includes the genes related to signaling and transcription, in particular Cs3g07510/AHP5 and orange1.1 t00294/ARR2 which are likely involved in cytokinin signaling. Cytokinin has been demonstrated to act in sink regulation of photosynthesis [39]. Specifically, cytokinin acts as both root-to-shoot and shoot-to-root signals and interacts with carbohydrate and nitrogen supplies to control the expression of photosynthesis genes. Given that expanding fruits can serve as a strong sink, it is conceivable that Cs3g07510 and orange1.1 t00294 may act to regulate cytokinin response, which then directs various cellular activities including driving the whole plant carbon and nitrogen metabolism so as to transport carbohydrates and amino acids to the developing fruits for sugar and acid metabolism.

Although our study has led to the identification of sugar-correlated genes in sweet orange fruits, this result on the large number of sugar-related genes needs to be interpreted with caution. With a Pcc cutoff +/−0.8, 56% of 7430 genes differentially expressed from 45 to 142 DPA have strong correlation with sugar (Additional file 1). Even with a higher Pcc cutoff (+/−0.9), we still obtained approximately 27% of genes that are highly correlated with sugar level (Additional file 1). Considering an early finding that more than 2500 genes are responsive to sugar in Arabidopsis [40], the number of 1995 sugar-correlated genes might be a closer approximation of genes potentially involved in either controlling sugar accumulation or responding to the increasing sugar status in fruit cells. Despite this, we should also consider the possibility that the large proportion (approximately 56% for Pcc of +/−0.8 or 27% for Pcc of +/−0.9) of genes showing strong correlations with sugar levels might be coincident with the similar pace in sugar accumulation and fruit growth and development. Our prior work has shown that among 7430 genes differentially regulated in any of the four varieties, 3145 genes are commonly regulated in all four varieties at 142 DPA compared to 45 DPA [14]. It is anticipated that these commonly regulated genes likely have conserved functions in fruit growth, development and metabolism in all four varieties during Stage II, such as in cell expansion and various aspects of metabolism. Indeed, analysis using Venn diagram showed that 1577 out of 1995 sugar-correlated genes are also commonly regulated (Fig. 1b). Therefore, it is conceivable that a considerable portion of 1995 sugar-correlated genes may function in cellular activities other than sugar and acid accumulation.

Given that mathematically the sugar/acid ratio is determined by sugar alone, acid alone and their relative levels, we had expected that many of the sugar/acid ratio-related genes should also strongly correlate with sugar or acid alone. However, to our surprise, we found that the majority of sugar/acid ratio-correlated genes are not correlated with either sugar or acid levels. There are at least two possibilities to explain this surprising finding. The first one is that most of these genes, if not all, might represent a unique group of genes which are involved in the control or sensing of sugar/acid ratio independently of controlling sugar alone or acid alone. Although the sugar/acid ratio seemed to exhibit an opposite pattern to acid content and a similar pattern with sugar content (Fig. 1A), none of the pairwise correlations among sugar level, acid level and the sugar/acid ratio in these four sweet orange varieties was statistically significant. Thus, this phenotypic variation among the four varieties seems to be consistent with this possibility. The second possibility is raised because mathematically the ratio can also be altered to a bigger difference even if sugar and acid levels are slightly altered in an opposite direction. In this case, some of the sugar/acid ratio-correlated genes would be expected to show weak correlations with the sugar or acid levels. Indeed, 20 such genes exhibit weak correlations with sugar (*p*-value <0.05), but only two genes have statistically significant (p-value < 0.05) weak correlations with the acid level (Additional file 2). If this is true, some of the sugar/acid ratio-related genes might have a minor role in micro-tuning the accumulation of either sugars or acids, in addition to their more promising roles in determining the sugar/acid ratio. Clearly, functional evidence will be critical for us to distinguish these two possibilities. It will also help to determine whether any of the sugar/acid ratio-related genes identified in this study is actually involved in the control of sugar/acid ratio rather than merely a gene expression response to the alterations in sugar/acid ratios during Stage II of fruit development. Whichever the possible mechanisms will be to explain our finding, work reported here has provided intriguing hypotheses for future testing using transgenic approaches.

## Conclusions

Our comparative transcriptome analysis has revealed a total of 72 genes that are highly correlated with the fruit sugar/acid ratio. However, very few of them correlate with sugar or acid contents, indicating that these genes may potentially be involved in maintaining fruit sugar/acid ratios and/or responding to the cellular sugar/acid ratio status. The majority of these sugar/acid ratio-related genes are predicted to be involved in regulatory functions such as transport, signaling and transcription or encode enzymes involved in metabolism. Results from gene coexpression network analysis indicate that half of these genes are organized into two regulatory modules unique to the sugar/acid ratio control. In summary, our analysis of orange transcriptomes provides an intriguing insight into the potentially novel genetic or molecular mechanisms controlling the sugar/acid ratio in fruits.

## Methods

### Data analysis

The sugar/acid ratios at 45 and 142 DPA for each of the four sweet orange (*C. sinensis* L. Osbeck) varieties with differing fruit acidity, Newhall, Xinhui, Bingtang and Succari, were derived from the sugar and acid levels reported in a prior manuscript [14]. Pairwise t-test was performed to determine the significant differences in sugar content, acid content and sugar/acid ratio between four varieties at 45 and 142 DPA. RNA sequencing-based transcriptome data deposited in the NCBI GEO database (Accession number GSE78046) was used for correlation study. RNA seq data has been processed and analyzed using the methods implemented in EdgeR, resulting in a total of 7430 genes that are differentially expressed between 45 and 142 DPA in any of the four varieties, with at least two-fold difference and an FDR cutoff of 0.05 as previously described [14].

### Pearson correlation analysis of sugar/acid ratios and gene expression levels

To identify the genes that show significant correlation between their expression levels and sugar/acid ratios, only those 7430 genes differentially expressed from 45 to 142 DPA in any of the four sweet orange varieties [14] were used for Pearson correlation coefficients (Pcc). Pcc are calculated between sugar/acid ratios and gene expression levels (which were first log2 transformed to normalize the data distribution) in a total of 24 samples (three biological replicates at each developmental stage for each variety), using an FDR cutoff of 0.05.

### GO analysis and sugar/acid ratio subnetwork construction and visualization

As described elsewhere [14], GO analysis for citrus genes was done by first predicting their most closely related Arabidopsis orthologs (based on TAIR Release 10) using the functional annotation website Mercator with a BLAST-Cutoff of 80 and then by assigning the GO terms for those Arabidopsis orthologs to the corresponding citrus genes. The gene coexpression network using 7430 differentially expressed genes was constructed using the WGCNA package in R [41], and a total of 10 different modules were derived as described elsewhere [14]. To construct various sugar/acid ratio subnetworks, the sugar/acid-correlated genes belonging to different modules were used as seed nodes to extract the gene coexpression network and the resulting gene-gene interactions were used to visualize the subnetworks using Cytoscape.

### Quantitative reverse transcription-PCR (qRT-PCR) analysis

qRT-PCR analysis was performed as described previously [14], using Cs1g05000-encoded actin gene (sense primer HZP14-L: TCCGTGACATGAAGGAGAAG; antisense primer HZP14-R: GCTCCAATGGTGATGATCTG) as a reference and specific primers for the following six genes: Cs1g03610 (QZQP201:TCTCGTACCGATGATTGCTC; QZQP202: GGCTGCTGGTTCAATATCAA), Cs5g03630 (QZQP205: TAATTGAAGCACAGCGAGGT; QZQP206: AGCCAAAGCAAGAGAGGAAC), Cs5g20010 (QZQP207: GAGAAACCGAGGCTACAAGC; QZQP208: TCTTCAGTTTCGGGACCAA), Cs5g24670 (QZQP209: CATGCTGACTTGGAATGCTT; QZQP210: AGGAAGCTTGCACTTATCCG), Cs6g16160 (QZQP211: GGAGAATGGGCTAATCGAAA; QZQP212: CTGTCTTCCCTGCATTAGCA), Cs7g32260 (QZQP215: GAGGAGCAACGTGTTCGAT; QZQP216: CGGAATATTAGCGACTGACG). The relative mRNA levels for each gene in Newhall at 45 DPA is set as 1.

## Additional files

Additional file 1: A list of genes correlated with the sugar level with Pcc = +/−0.8 and 0.9, respectively. (XLSX 286 kb)

Additional file 2: Correlation of sugar/acid raio-correlated genes with sugar or acid levels. (XLSX 17 kb)

## Abbreviations

AHP5: Arabidopsis histidine phosphotransfer protein 5
ARR2: Arabidopsis response regulator 2
CNGC1: Cyclic nucleotide gated channel 1
DPA: Days post anthesis
FDR: False discovery rate
GO: Gene ontology
MDH2: Malate dehydrogenase 2
Pcc: Pearson correlation coefficients
PGI: Glucose-6-phosphate isomerase or phosphoglucose isomerase
qRT-PCR: Quantitative reverse transcription-PCR
RLK: Receptor-like kinases
WGCNA: Weighted Gene Coexpression Network Analysis
